# Respiratory Exposure to Agriculture Dust Extract Alters Gut Commensal Species and Key Metabolites in Mice

**DOI:** 10.1002/jat.4808

**Published:** 2025-05-08

**Authors:** Meli’sa S. Crawford, Arzu Ulu, Briana M. Ramirez, Alina N. Santos, Pritha Chatterjee, Vinicius Canale, Salomon Manz, Hillmin Lei, Sarah Mae Soriano, Tara M. Nordgren, Declan F. McCole

**Affiliations:** 1 School of Medicine, Division of Biomedical Sciences, University of California, Riverside, California, USA; 2 School of Environmental and Biological Sciences, Department of Animal Sciences, Rutgers-New Brunswick, New Brunswick, New Jersey, USA; 3 Department of Biochemistry and Molecular Biology, University of California, Riverside, California, USA; 4 Bishop Gorman High School, Las Vegas, Nevada, USA; 5 Department of Pediatrics, University of Nebraska Medical Center, Omaha, Nebraska, USA

**Keywords:** agricultural pollution, *Akkermansia muciniphila*, gut microbiome, gut–lung axis, intestinal barrier function

## Abstract

Exposure to agricultural dust containing antimicrobial-resistant pathogens poses significant health risks for workers in animal agriculture production. Beyond causing severe airway inflammation, pollutants are linked to intestinal diseases. Swine farm dust is rich in ultrafine particles, gram-positive and gram-negative bacteria, and bacterial components such as lipopolysaccharides (LPS; endotoxins). In our previous study, we demonstrated that intranasal exposure of male and female C57BL/6J mice to 12.5% hog dust extract (HDE, containing 22.1–91.1 EU/mL) for 3 weeks resulted in elevated total cell and neutrophil counts in bronchoalveolar lavage fluid and increased intestinal permeability compared to saline controls. Now, we report that 16S and metagenomic analyses of Week 3 stool samples from HDE-treated mice indicate a reduced abundance of the beneficial species *Akkermansia muciniphila* and *Clostridium sp. ASF356* and *Lachnospiraceae* bacterium. Bacterial alpha diversity showed increased species evenness in fecal samples from HDE-treated mice (Pielou’s evenness, *p* = 0.047, *n* = 5–6/group). Metabolomic analysis also indicated significant reductions in key metabolites involved in energy metabolism, including riboflavin (*p* = 0.027, *n* = 11) and nicotinic acid (*p* = 0.049, *n* = 11), as well as essential amino acids, such as inosine (*p* = 0.043, *n* = 11) and leucine (*p* = 0.018, *n* = 11). While HDE exposure does not robustly alter overall microbial abundance or community structure, it leads to specific reductions in beneficial bacterial species and critical metabolites necessary for maintaining intestinal homeostasis by supporting energy metabolism, gut barrier function, microbiota balance, and immune regulation. The results of this study underscore the potential risks for gut health posed by inhalation of agricultural dust.

## 1 | Introduction

Decades of research have shown that air pollution, such as ozone and particulate matter (PM), contribute to the development of respiratory, cardiovascular (De Filippis et al. 2024), and gastrointestinal diseases (Chiu et al. 2020; Claus et al. 2016; Pritchett et al. 2022). While many sources of pollution have been identified and linked to human disease, the impact of agricultural pollutants on human health is understudied. Pollution produced by animal feeding operations (AFO) poses many health risks for farmers and farmworkers, particularly those who work on swine farms. These risks include exposure to significant amounts of dust that may contain mold, bacteria, and significant amounts of ammonia and PM (Wang et al. 2021).

Inhalation of these pollutants can promote lung inflammation through several immune mechanisms, which may lead to systemic effects, including intestinal barrier dysfunction (Crawford et al. 2024) and gut dysbiosis (Mutlu et al. 2011). Although there are few studies demonstrating how airborne particles from farms, especially bioaerosols from livestock operations, can influence gut microbial diversity, studies have linked environmental PM exposure to altered gut microbial composition and increased colitis severity (Chen et al. 2019). Studies examining antimicrobial-resistant genes (resistomes) in swine feces and farm dust also suggest that bacteria in dust may contribute to antibiotic resistance in both the lungs and the gut (Luiken et al. 2020), further linking agricultural dust to alterations in microbial communities.

Our previous study investigated the detrimental effects of inhaled hog dust extract (HDE) on both airway and intestinal health, revealing a complex interaction between respiratory exposure and gastrointestinal pathophysiology, emphasizing the gut–lung axis in disease progression (Crawford et al. 2024). Previous shotgun pyrosequencing of HDE (Boissy et al. 2014) confirmed the presence of gram-positive and gram-gram-negative bacteria and lipopolysaccharides (LPS; endotoxins). The inflammatory effects examined in mice following HDE exposure may be the result of LPS, which is a potent inflammatory agent (Crawford et al. 2024). Moreover, inhalation of endotoxins can increase gut permeability (McQuade et al. 2023). Consequently, this could allow HDE, HDE byproducts, and/or pathogenic bacteria to cross the intestinal barrier, thereby further contributing to dysbiosis and gut inflammation (Li et al. 2023). Prior animal studies have indicated that exposure to HDE can exacerbate chronic airway inflammation, as evidenced by elevated levels of immune cells (e.g., neutrophils) in bronchoalveolar lavage fluid (Crawford et al. 2024; Romberger et al. 2016). However, the effects of agriculture pollutants on the gut flora and physiology remain largely unexplored.

In this study, we hypothesize that acute intranasal exposure to aqueous extracts of hog dust will induce intestinal inflammation and disrupt gut barrier function through alterations in gut microbial communities. By examining changes in gut microbiota composition in an in vivo intranasal exposure model, we aim to elucidate the systemic effects of agricultural dust exposure. Our findings show alterations in beneficial intestinal species and suggest a link between respiratory exposure to agricultural dust and intestinal outcomes, therefore, highlighting the need for protective measures for agricultural workers.

## 2 | Materials and Methods

### 2.1 | Hog Dust Extract (HDE)

Dust extracts were prepared as previously described (Wyatt et al. 2020). Briefly, settled dusts were collected from swine confinement facilities and stored at −20°C. To prepare 100% aqueous hog dust extract, 5-g dust was suspended in 50 mL of balanced salt solution for 1 h, then filtered and sterilized through a 0.22-mm filter (Ulu et al. 2021). The 100% aqueous dust solution was aliquoted and stored at −20°C until used. A 12.5% HDE solution was prepared for instillation by diluting 100% HDE (containing 22.1–91.1 EU/mL) in sterile phosphate buffered saline (PBS). Study animals were terminally anesthetized with isoflurane (Catalog # NDC-11695-6776-2, Henry Schein, Melville, NY, USA). All procedures were approved by the University of California, Riverside, (UCR) Institutional Animal Care and Use Committee (McCole 20190032; Nordgren; 20200014).

### 2.2 | Animal Models

This study is a continuation of published work from our laboratory using 8-week-old C57BL6/J mice (*n* = 11) randomly divided into two treatment groups that received either a 50-μL intranasal dose of PBS or 12.5% HDE, containing 22.1–91.1 EU/mL, for daily for 3 weeks (five consecutive days per week) under light sedation (repetitive exposure) as previously described (Poole et al. 2009). Mice were acclimatized for 1 week upon arrival before experimentation and were maintained on a 12:12-h light:dark cycle. Water and standard rodent chow were ad libitum. Body weight was measured daily, and fecal samples were collected weekly.

### 2.3 | Fecal Microbiome Analyses

Stool samples were collected at 3 weeks and sent to the University of California, San Diego (UCSD) Microbiome Core. The UC San Diego Microbiome Core performed nucleic acid extractions utilizing previously published protocols (https://microbiomejournal.biomedcentral.com/articles/10.1186/s40168-021-01083-0). Briefly, samples were purified using the MagMAX Microbiome Ultra Nucleic Acid Isolation Kit (Thermo Fisher Scientific, USA) and automated on KingFisher Flex robots (Thermo Fisher Scientific, USA). Blank controls and mock communities (Zymo Research Corporation, Tustin, CA, USA) were included and carried through all downstream processing steps. DNA was quantified using a PicoGreen fluorescence assay (Thermo Fisher Scientific, Waltham, MA, USA), and metagenomic libraries were prepared with the KAPA HyperPlus kit (Roche Diagnostics, Basel, Switzerland) and automated on EpMotion automated liquid handlers (Eppendorf, Hamburg, Germany). Sequencing was performed on the Illumina NovaSeq 6000 sequencing platform with paired-end 150-bp cycles at the Institute for Genomic Medicine (IGM), UC San Diego. The 16S rRNA gene amplification was performed according to the Earth Microbiome Project protocol (https://microbiomejournal.biomedcentral.com/articles/10.1186/s40168-021-01083-0). Briefly, Illumina primers with unique forward primer barcodes were used to amplify the V4 region of the 16S rRNA gene (515fB-806r, https://journals.asm.org/doi/10.1128/msystems00009-15). Amplification was performed with single reactions per sample (https://www.future-science.com/doi/10.2144/btn-2018-0192), and equal volumes of each amplicon were pooled for sequencing. The 16S libraries were sequenced at the UC San Diego Institute for Genomic Medicine on the Illumina MiSeq sequencing platform with paired-end 150 bp cycles. The UCSD Microbiome Core performed sample extractions and library preparation utilizing protocol and primers published on the Earth Microbiome Project website. This publication includes data generated at the UC San Diego IGM Genomics Center utilizing an Illumina NovaSeq 6000 that was purchased with funding from a National Institutes of Health SIG grant (#S10 OD026929). The Bioinformatics Core at the UCR implemented QIIME analyses to analyze microbial taxonomy from Week 0 and Week 3 fecal samples as previously described. Subsequently, microbial differences were examined using linear discriminant analysis of effect size (LEfSe) analyses implemented within the online Galaxy module (http://huttenhower.sph.harvard.edu/galaxy/) explained by Segata et al. (2011). Relative abundance percentages were then calculated from taxonomic data generated from QIIME.

#### 2.3.1 | Internal Transcribed Spacer (ITS) Analysis

ITS gene amplification was performed according to the Earth Microbiome Project protocol (https://www.nature.com/articles/nature24621). Briefly, Illumina primers with unique reverse primer barcodes were used to amplify the ITS1 region of the fungal ITS spacer gene (ITS1f-ITS2, https://journals.asm.org/doi/10.1128/mSystems.00009-15). Amplification was performed with single reactions per sample (https://www.future-science.com/doi/10.2144/btn-2018-0192), and equal volumes of each amplicon were pooled for sequencing. ITS libraries were sequenced at the UC San Diego Institute for Genomic Medicine on the Illumina MiSeq nano sequencing platform with paired-end 150-bp cycles.

### 2.4 | Central Carbon Metabolomics

Whole blood (1 mL) was collected from the inferior vena cava from PBS controls and HDE-treated mice. Serum was collected by centrifuging the whole blood (Thermo Fisher Scientific, Sorvall Legend Micro 21R Centrifuge, Waltham, MA, USA) at 4°C for 15 min. Serum samples were analyzed by the UCR Metabolomics Core (Riverside, CA, USA).

#### 2.4.1 | Sample Preparation

Approximately 100 μL of serum was transferred to glass vials, and four volumes of methanol were added. A quick vortex was conducted to mix samples. Samples were extracted by sonication on ice for 5 min, followed by 60 min at −80°C. The samples were centrifuged at 4°C for 30 min and 3000 g. Supernatants were collected and pipetted into LC–MS vials. A quality control (QC) sample was constituted by combining equal volumes of all samples and used to monitor system stability.

#### 2.4.2 | LC–MS for Central Carbon Metabolism

Targeted metabolomics of polar, primary metabolites was performed at the UCR Metabolomics Core Facility as described previously. Briefly, analysis was performed on a TQ-XS triple quadrupole mass spectrometer (Waters, Milford, MA, USA) coupled to an I-class UPLC system (Waters, Milford, MA, USA). Separations were carried out on a ZIC-pHILIC column (2.1 × 150 mm, 5 μM) (MiliporeSigma, Burlington, MA, USA). The mobile phases were (A) water with 15-mM ammonium bicarbonate adjusted to pH 9.6 with ammonium hydroxide and (B) acetonitrile. The flow rate was 200 μL/min, and the column was held at 50°C. The injection volume was 2 μL. The gradient was as follows: 0 min, 90% B; 1.5 min, 90% B; 16 min, 20% B; 18 min, 20% B; 20 min, 90% B; and 28 min, 90% B. The MS was operated in multiple reaction monitoring mode (MRM). Source and desolvation temperatures were 150°C and 600°C, respectively. Desolvation gas was set to 1100 I/h and cone gas to 150 L/h. Collision gas was set to 0.15 mL/min. All gases were nitrogen except the collision gas, which was argon. Capillary voltage was 1 kV in positive ion mode and 2 kV in negative ion mode. A QC sample, generated by pooling equal aliquots of each sample, was analyzed periodically to monitor system stability and performance. Samples were analyzed in random order.

### 2.5 | DNA Extraction and qPCR

DNA from luminal contents was extracted using the Qiagen DNeasy PowerSoil Kit (Catalog # 47014; Hilden, Germany) according to the manufacturer’s protocol. iQ SYBR Green Supermix (Bio-Rad, Hercules, CA, USA) was used for real-time quantitative PCR on a C1000 Thermal cycler equipped with a CFX96 real-time PCR system and the Bio-Rad CFX Manager 3.1 Software. The real-time PCR included an initial enzyme activation step (3 min, 95°C) followed by 45 cycles consisting of a denaturing (95°C, 10 s), an annealing (53°C–60°C, 10 s), and an extending (72°C, 10 s) step. The PCR included the following steps for each primer: *Akkermansia muciniphila* (*A. muciniphila*; *forward:* 5′-CAGCACGTGAAGGTGGGGAC-3′ *and reverse:* 3′-CCTTGCGGTTGGCTTCAGAT-5′) and Universal primer (forward: 5′-CTCCTACGGGAGGCAGCAG-3′and reverse: 5′-TTACCGCGGCTGCTGGCAC-3′). Results were analyzed by the ΔΔCT method.

### 2.6 | Statistical Analyses

Data were analyzed by unpaired *t* test followed by Mann–Whitney post hoc tests. Statistical significance was considered as *p* < 0.05 for all analyses. Data were analyzed using GraphPad Prism 9 software (San Diego, CA, USA).

#### 2.6.1 | Data Analysis for Central Carbon Metabolism

Targeted data processing was performed with the open-source Skyline software (MacLean et al. 2010) for peak area integration. A panel of 195 metabolites with known retention times and transitions was used as a reference (van Vliet et al. 2021). After metabolite identification and relative quantification, we used a UCR Metabolomics Core proprietary tool, an HTML-based solution for data handling. Principal components analysis (PCA) was performed with the metabolomics data (Giri 2004; Johnson and Wichern 2007) using princomp (Mardia et al. 1979) or prcomp (Mardia et al. 1979) package in R. Heatmaply package (Galili et al. 2018) in R was utilized for the calculation and visualization of heat maps using *z* scores (van den Berg et al. 2006) calculated by subtracting mean relative abundance values in each row for each metabolite from relative abundance values and dividing this number by the standard deviation in each row for each metabolite. For pairwise comparison between treatments. Welch’s *t* test (Welch 1947) was applied to Log2 transformed data (Bennett 1962). *p* values were corrected using Benjamini and Hochberg’s (1995) correction. Volcano plots (Hur et al. 2013) for displaying the *p* or *q* values of statistically significant polar metabolites on the *y* axis and fold changes of polar metabolites between two different groups on the *x* axis. The significantly different metabolites (*p* or *q* value < 0.05) were displayed in boxplots (Moses 1987), bar plots, or violin plots.

## 3 | Results

### 3.1 | Alpha Diversity Increased in Repetitive HDE Group

**FIGURE 1 |:**
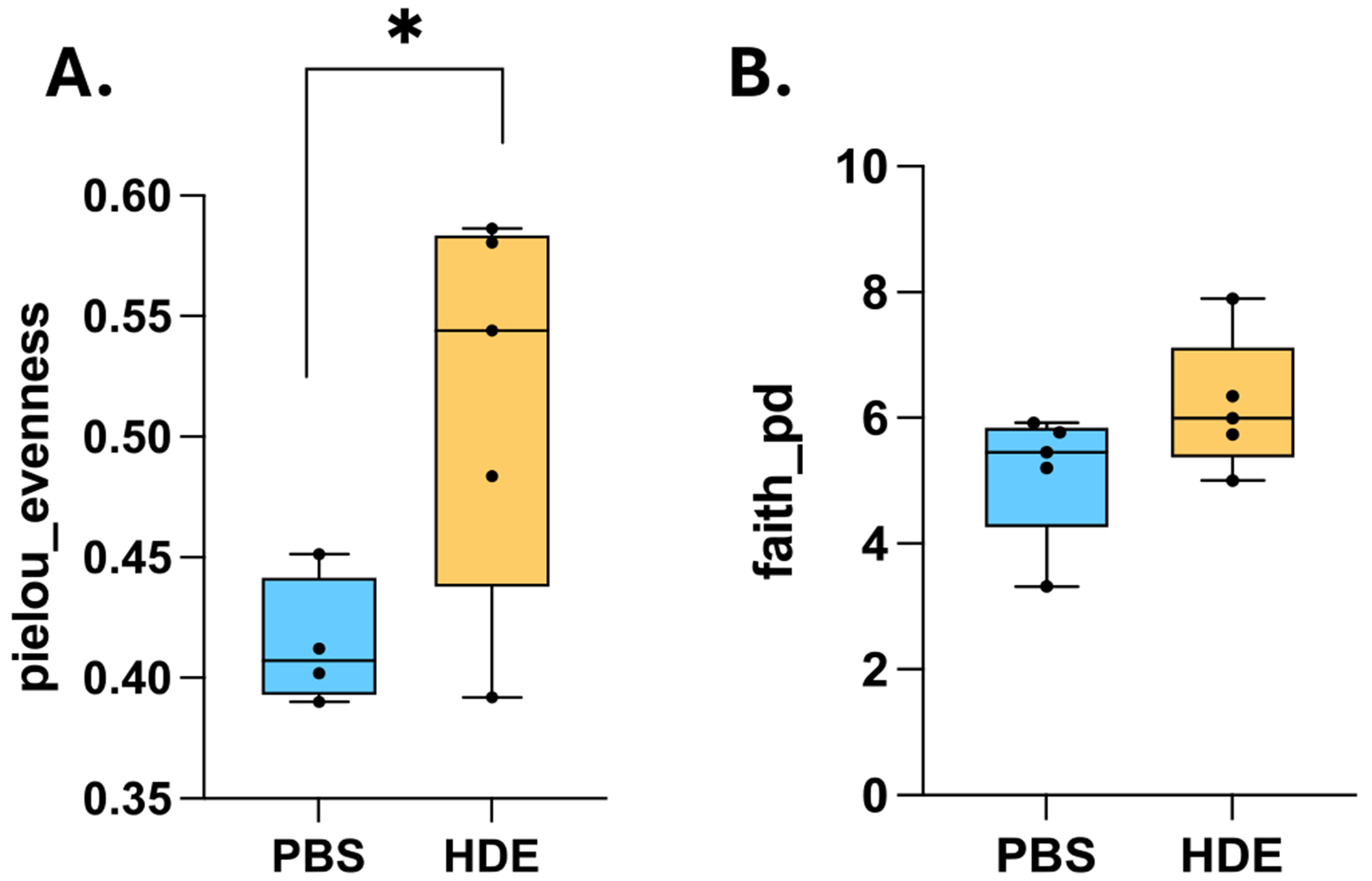
Week 3 HDE stool samples have higher evenness and modest biodiversity: (A) Pielou’s evenness shows that species in HDE samples are present in similar proportions in comparison to saline control samples (Kruskal–Wallis pairwise, *p* = 0.047, *n* = 5/group) thereby indicating higher species diversity following dust exposure. (B) Faith’s PD shows a suggestive increase in biodiversity in HDE samples (Kruskal–Wallis pairwise, *p* = 0.174, *n* = 5/group).

**FIGURE 2 |:**
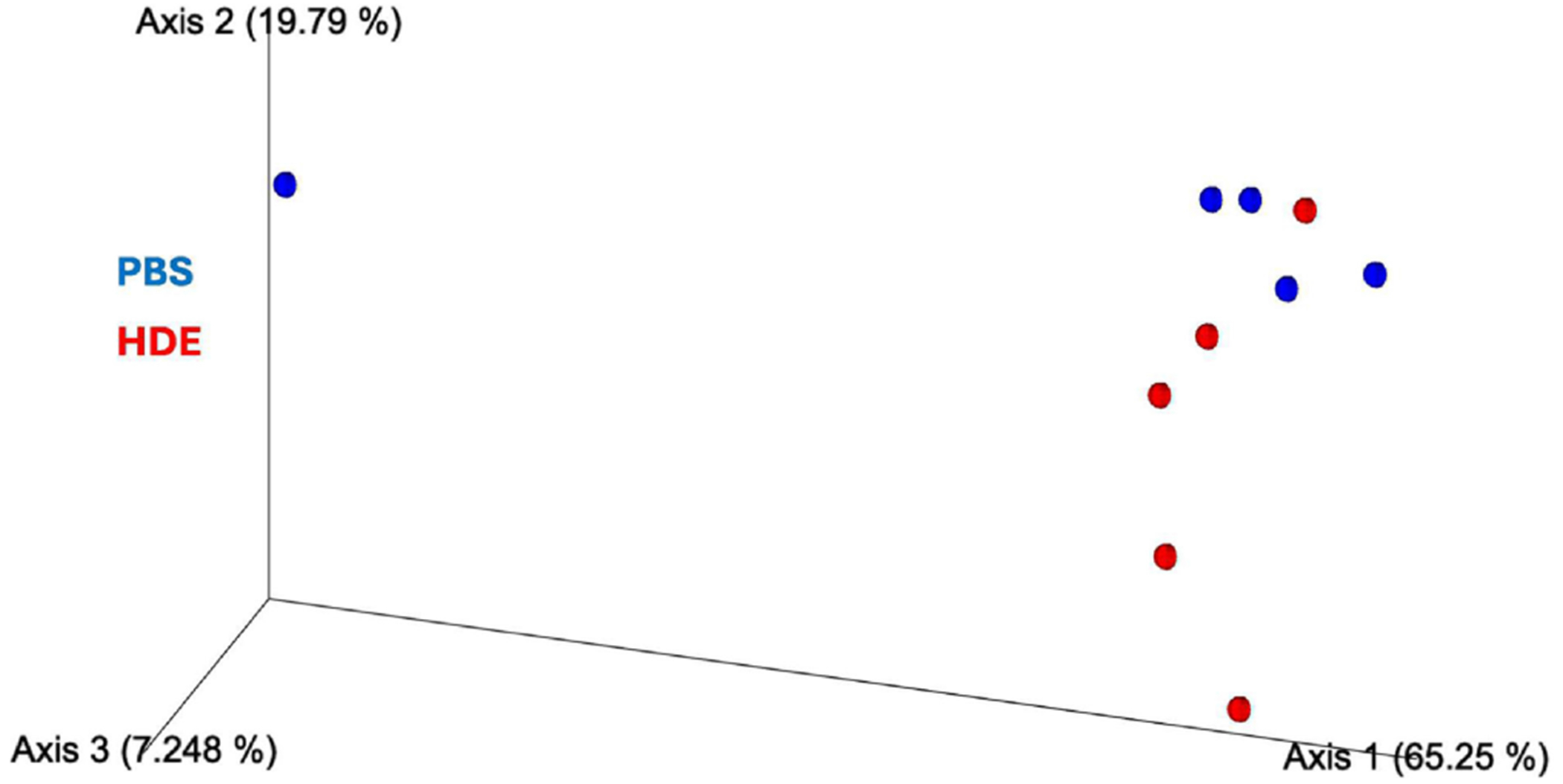
PBS and HDE Week 3 samples have similar microbial community composition: Unweighted unifrac distances show no dissimilarity between PBS (blue) and HDE (red) Week 3 samples (PERMANOVA, *p* = 0.565; *n* = 5/group). QIIME2 was utilized to compute alpha and beta diversity metrics and generate emperor plots for PBS and HDE Week 3 samples.

The differences in alpha diversity, a measure of the richness and evenness of a bacterial sample within a habitat, between PBS controls and HDE groups were compared using Pielou’s evenness indices and Faith’s phylogenetic diversity (Faith’s PD) metric. Interestingly, evenness was higher in the repetitive HDE group than in saline controls (Figure 1A; Kruskal–Wallis pairwise, *p* = 0.047, *n* = 5/group). Moreover, a suggestive increase in biodiversity was observed in the repetitive HDE group as measured by Faith’s PD (Figure 1B; Kruskal–Wallis pairwise, *p* = 0.174, *n* = 5/group). The Unweighted Unifrac Distance metric assessed beta diversity and examined community composition, and no dissimilarities between PBS and HDE Week 3 samples fecal samples were shown (Figure 2; PERMANOVA, *p* = 0.565; *n* = 5/group).

### 3.2 | HDE Exposure Did Not Alter the Relative Abundance of Intestinal Bacteria in Mice

**FIGURE 3 |:**
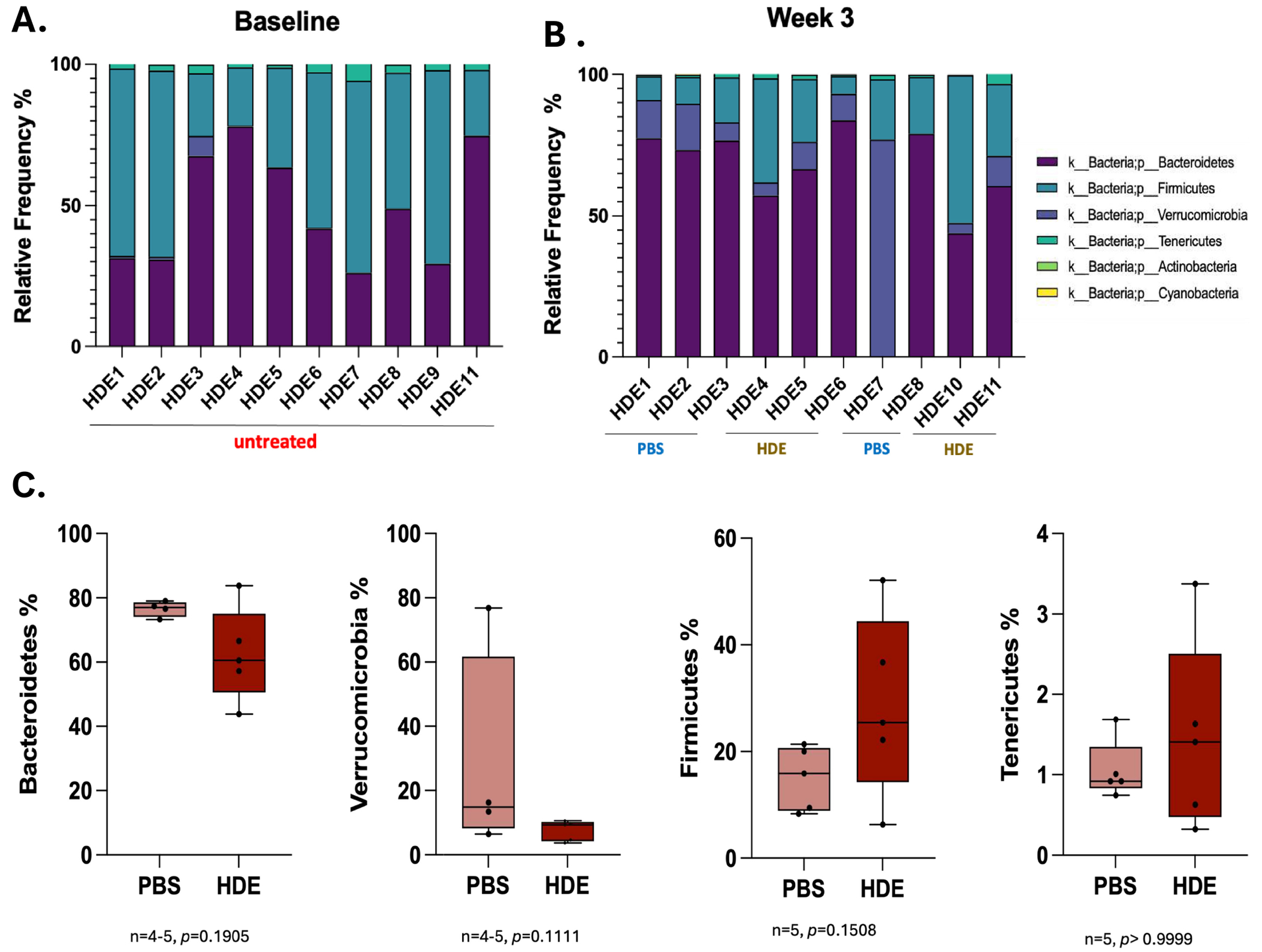
Microbial composition shows no significant phylum level differences at Week 3: Illumina sequencing and QIIME2 analyses were used to analyze microbial taxonomy from (A) baseline and (B) Week 3 stool samples. Relative frequency of gut microbiota at phylum level in stool samples of each group at baseline and Week 3. (C) Relative frequency of phyla examined in Week 3 stool samples: Bacteroidetes (unpaired *t* test, *p* = 0.1905, *n* = 4–5), Verrucomicrobia (unpaired *t* test, *p* = 0.1111, *n* = 4–5), Firmicutes (unpaired *t* test, *p* = 0.1508, *n* = 5), and Tenericutes (unpaired *t* test, *p* > 0.05, *n* = 5).

To assess the impact of agricultural pollutants on host gut microbial abundance, we collected fecal samples at baseline and Day 15 from saline controls and HDE-exposed mice. Repetitive HDE exposure for 15 days did not significantly alter microbial abundance (Figure 3A,B). However, at taxonomic level 2 (phyla), data show a suggestive reduction in *Verrucomicrobia* (unpaired *t* test, *p* = 0.1111, *n* = 4–5/group) and an increased trend in *Firmicutes* (unpaired *t* test, *p* = 0.1508, *n* = 5) in HDE-treated mice in comparison to PBS controls (Figure 3C), without reaching significance.

### 3.3 | HDE Treatment Appears to Reduce the Abundance of *A. muciniphila* Species in Mice

**FIGURE 4 |:**
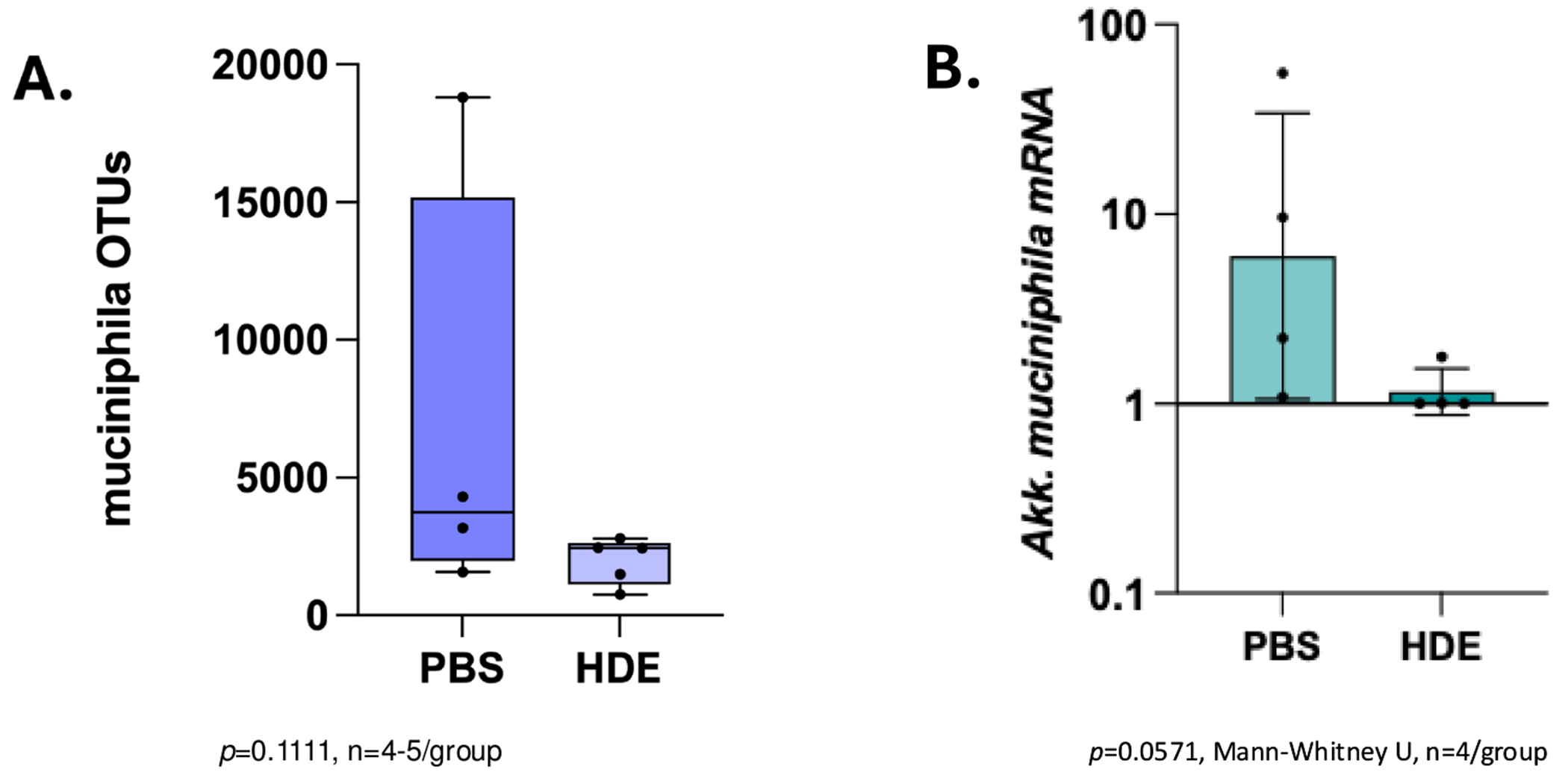
HDE-reduced *Akkermansia muciniphila* expression in the colon. (A) Microbial species level analysis (16S rRNA) revealed a partial decrease in *A. muciniphila* abundance in HDE-treated mice (unpaired *t* test, *p* = 0.1111, *n* = 4–5/group). (B) Gene expression of *A. muciniphila* examined in proximal colon luminal contents by qPCR was reduced in HDE-exposed mice (unpaired *t* test, *p* = 0.0571; *n* = 4/group).

Examination of microbial analysis at the species level (***Taxonomic level 7***) by 16S rRNA sequencing revealed a partial but statistically insignificant reduction of the beneficial species, *A. muciniphila* (Figure 4A; unpaired *t* test, *p* = 0.1111, *n* = 4–5/group). Next, we investigated the effects of HDE on the mRNA expression of key microbial species in luminal contents. *A. muciniphila* showed a marginal reduction in proximal colon luminal contents of HDE-exposed mice compared to saline controls (Figure 4B; unpaired *t* test, Mann–Whitney U, *p* = 0.0571, *n* = 4/group).

### 3.4 | Metagenomic Analysis Identified a Reduction in Beneficial Species Following HDE Exposure

**FIGURE 5 |:**
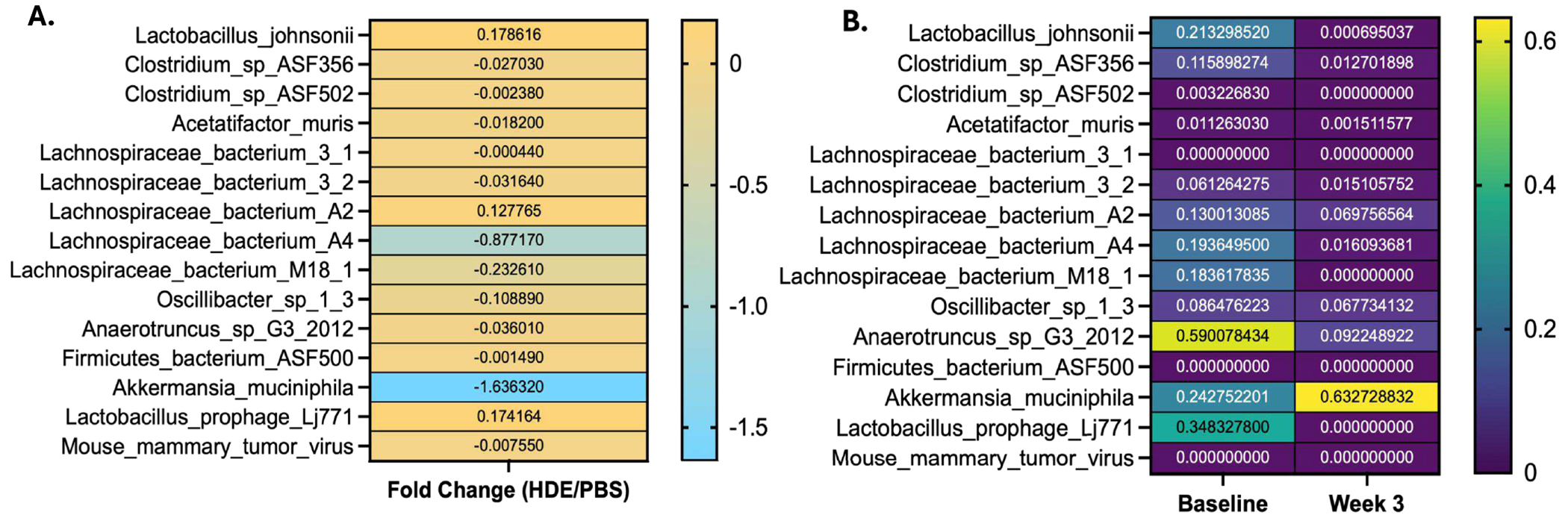
Metagenomic data show HDE exposure decreased the abundance of *Akkermansia muciniphila* and other beneficial species in feces. (A) Heatmap of taxa characterized in Week 3 fecal samples from saline controls and mice exposed to HDE. (B) Heatmap of taxa found in baseline and Week 3 fecal samples from HDE-exposed mice.

**FIGURE 6 |:**
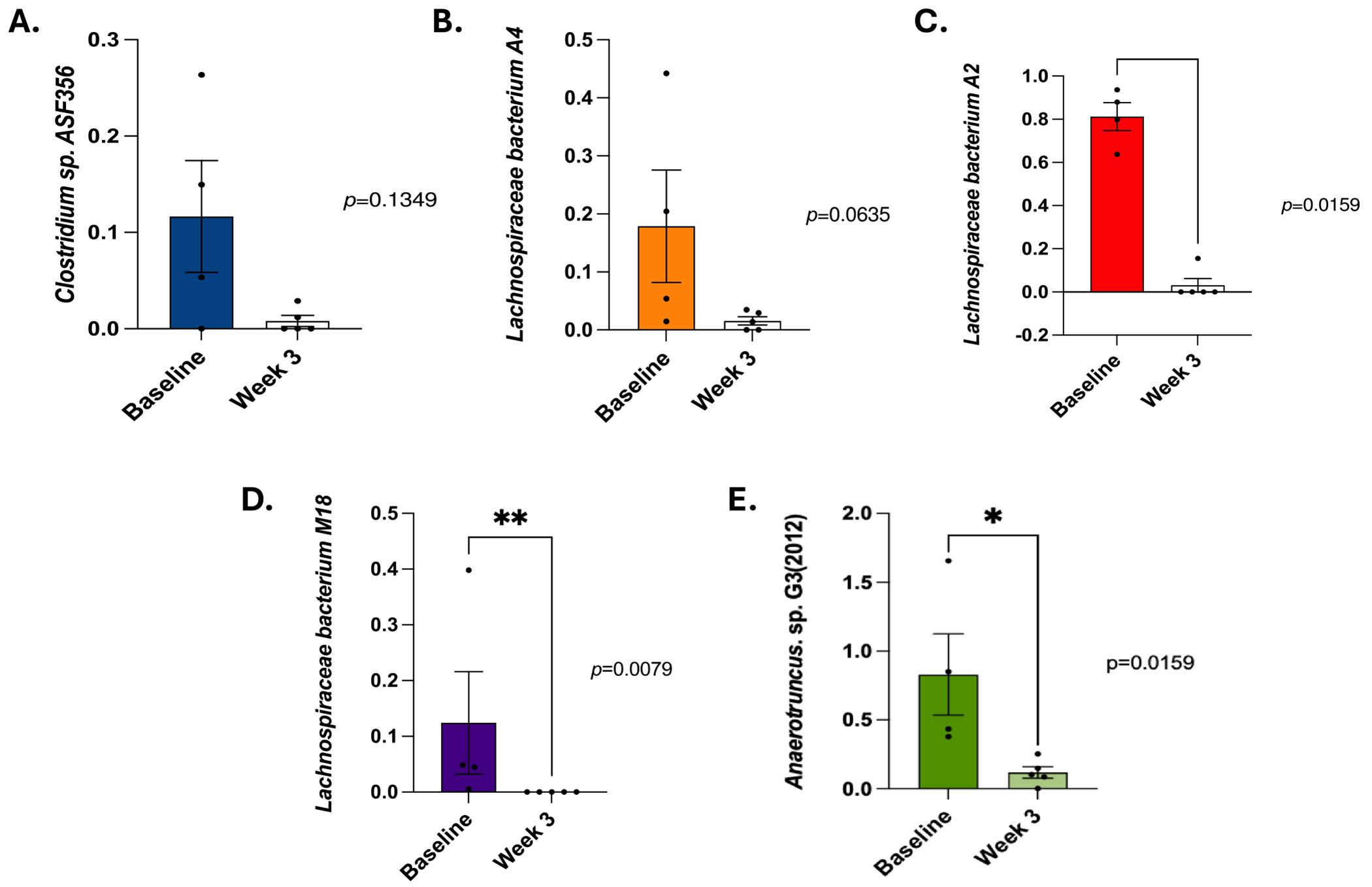
Focused metagenomic analysis shows reduced expression of beneficial species in HDE-exposed mice. Metagenomic sequencing shows a partial but statistically insignificant reduction of *Clostridium species ASF356* (A; unpaired *t* test, *p* = 0.1349, *n* = 5–6/group), *Lachnospiraceae bacterium A4* (B; unpaired *t* test, *p* = 0.0635, *n* = 5–6/group), while there was a significant reduction in *Lachnospiraceae bacterium A2* (Figure 5C; unpaired *t* test, *p* = 0.0159, *n* = 5–6/group) and *Lachnospiraceae bacterium M18* (Figure 5D; unpaired *t* test, *p* = 0.0079, *n* = 5–6/group) and *Anaerotruncus species G3* (Figure 5E; unpaired *t* test, *p* = 0.0159, *n* = 5–6/group).

To further investigate the microbiome and characterize the bacterial genome following exposure to HDE, metagenomic next-generation sequencing was conducted on fecal samples collected from saline controls and repetitive HDE study animals. Metagenomic sequencing revealed that fold change values, calculated by normalizing HDE-treated samples to PBS control (with PBS serving as the baseline reference), showed a reduction in *A. muciniphila* expression in fecal samples at 3 weeks post exposure (Figure 5A). Additionally, analysis of baseline (pre-HDE exposure) metagenomic data compared to HDE Week 3 data revealed an increase in *A. muciniphila* abundance (Figure 5B). The observed increase in *A. muciniphila* from baseline to Week 3 in HDE-treated mice was followed by a relative reduction compared to PBS controls. We next examined individual species that were altered at Week 3 HDE exposure. Metagenomic sequencing showed a partial reduction of *Clostridium species ASF356* (Figure 6A; unpaired *t* test, *p* = 0.1349, *n* = 5–6/group), *L. bacterium M8* (Figure 6B; unpaired *t* test, *p* = 0.0635, *n* = 5–6/group), while there was a significant reduction in *Anaerotruncus species G3* (Figure 6C; unpaired *t* test, *p* = 0.0159, *n* = 5–6/group), *L. bacterium A2* (Figure 6D; unpaired *t* test, *p* = 0.0159, *n* = 5–6/group), and *L. bacterium M18* (Figure 6E; unpaired *t* test, *p* = 0.0079, *n* = 5–6/group). These data indicate shifts in microbial communities associated with the production of metabolites important for maintaining barrier integrity.

### 3.5 | ITS Sequencing Analysis Showed No Significant Effect of HDE Exposure on Fungal Communities

**FIGURE 7 |:**
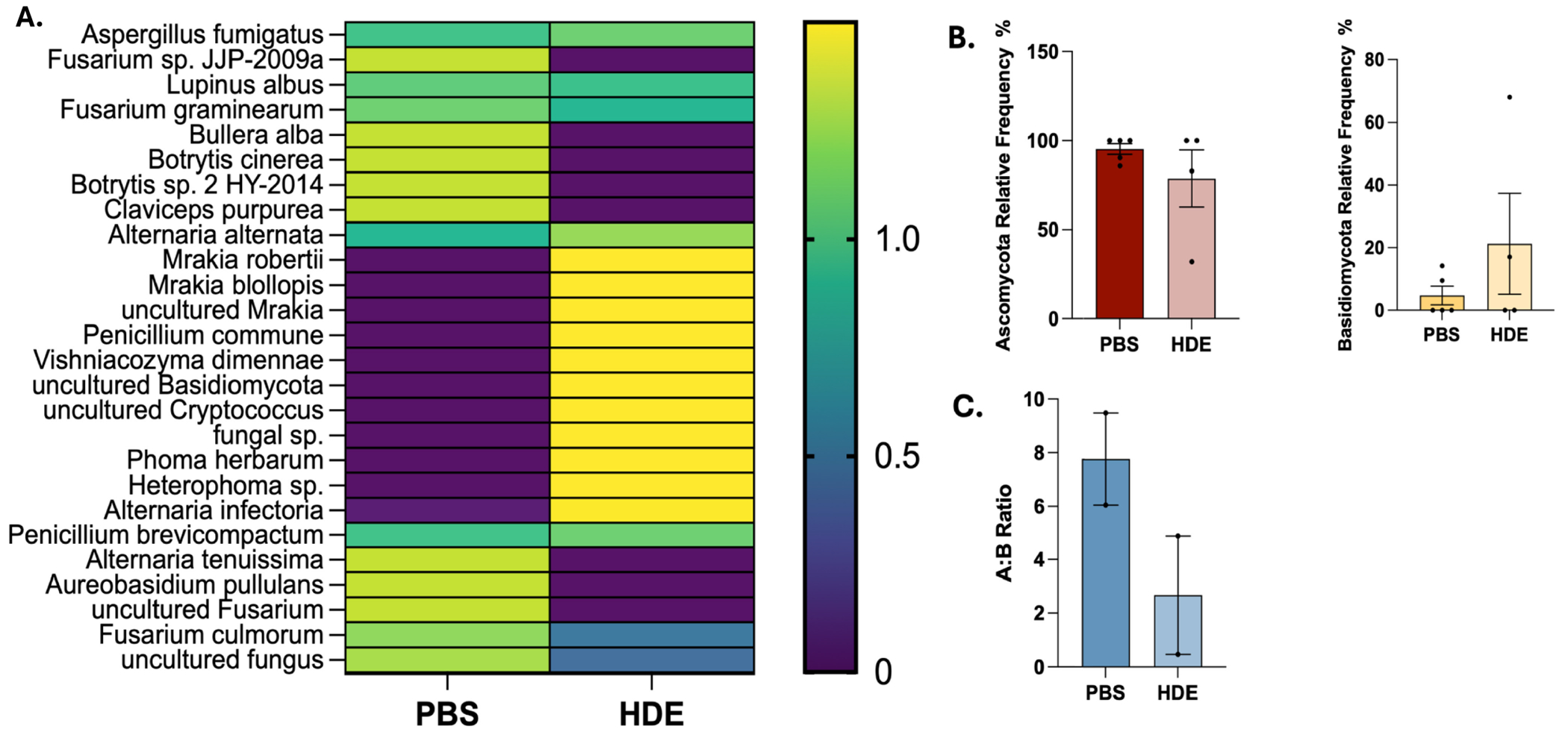
ITS sequencing analyses show minor differences in fungal communities: (A) Heat map shows Week 3 fungal taxonomy of PBS and HDE groups. The rows represent operational taxonomic units (OTUs) or strains of fungi within the groups, and the columns represent each group. (B) Relative frequency of dominant divisions, *Ascomycota* and *Basidomycota*, respectively. (C) Ratio of *Ascomycota* and *Basidomycota* found in Week 3 fecal samples from saline controls and HDE-exposed mice (unpaired *t* test, *p* = 0.2095, *n* = 5–6/group).

**FIGURE 8 |:**
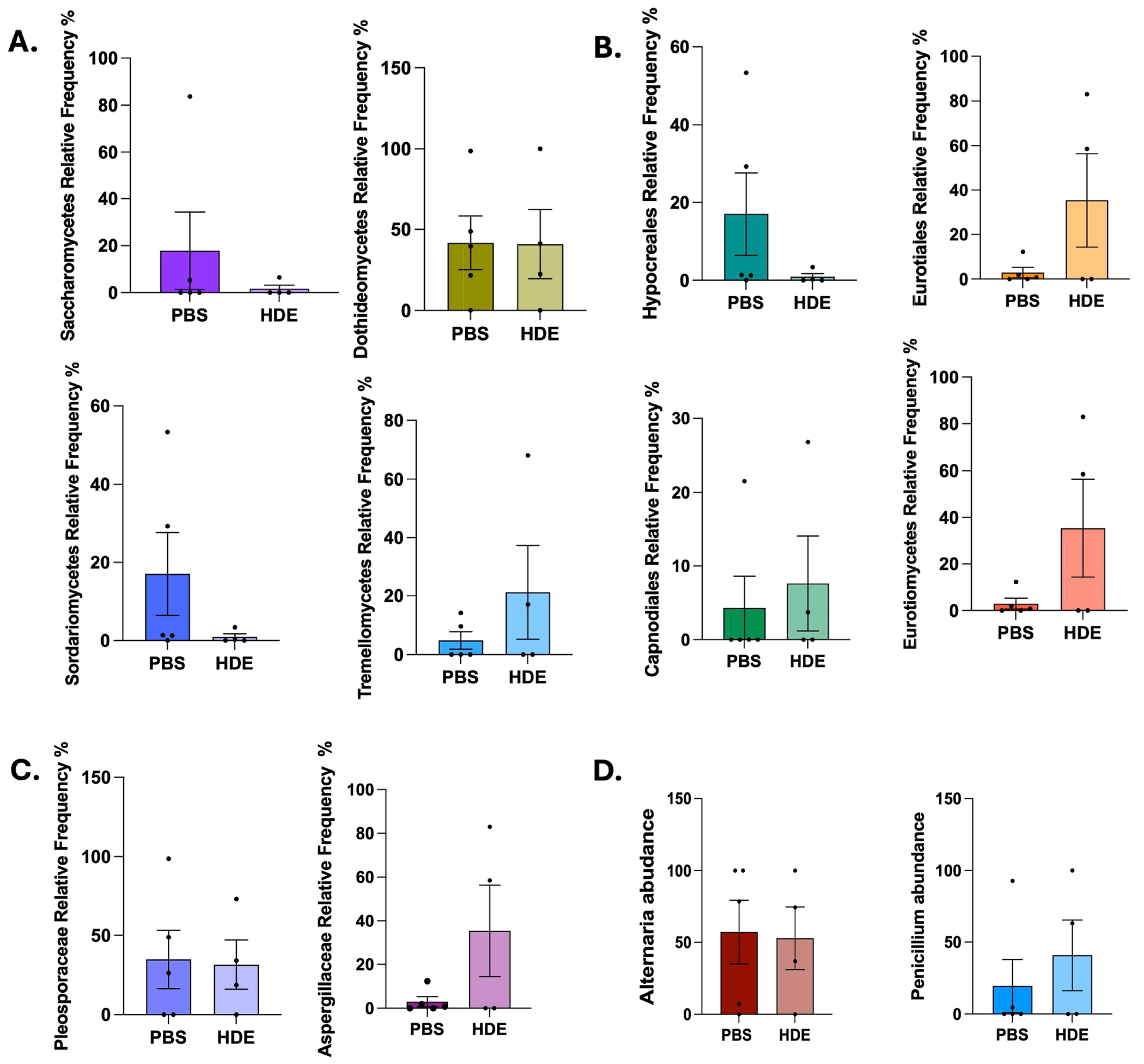
Relative frequency of ITS characterization at Week 3 shows minor differences between groups: ITS sequencing shows fungal relative frequency in fecal samples from PBS control and HDE-exposed mice at Week 3. (A; *Class*): Saccharomycetes (unpaired *t* test, *p* = 0.4167, *n* = 4–5/group), Dothideomycetes (unpaired *t* test, *p* = 0.9748, *n* = 4–5/group), Sordariomycetes (unpaired *t* test, *p* = 0.2221, *n* = 4–5/group), Tremellomycetes (unpaired *t* test, *p* = 0.2934, *n* = 4–5/group), and Eurotiomycetes (unpaired *t* test, *p* = 0.1260, *n* = 4–5/group). (B; *Order*): Hypocreales (unpaired *t* test, *p* = 0.2221, *n* = 4–5/group), Eurotiales (unpaired *t* test, *p* = 0.1260, *n* = 4–5/group), and Capnodiales (unpaired *t* test, *p* = 0.6700, *n* = 4–5/group). (C; *Family*) Pleosporaceae (unpaired *t* test, *p* = 0.8986, *n* = 4–5/group) and Aspergillaceae (unpaired *t* test, *p* = 0.1260, *n* = 4–5/group). (D; *Genus*) Alternaria (unpaired *t* test, *p* = 0.8954, *n* = 4–5/group) and Penicillium (unpaired *t* test, *p* = 0.5015, *n* = 4–5/group).

Having identified HDE-induced changes in bacterial species, we next characterized gut fungal communities following HDE exposure. ITS sequencing was conducted on fecal samples collected from saline and repetitive HDE study animals. HDE exposure did not significantly impact overall fungal communities (Figure 7, heatmap), and deeper analysis at the Class, Order, Family, and Genus levels did not show any alterations in fungal abundance (Figure 8A–D, *p* < 0.05).

### 3.6 | HDE Exposure Alters Metabolites Involved in Central Carbon Metabolism

**FIGURE 9 |:**
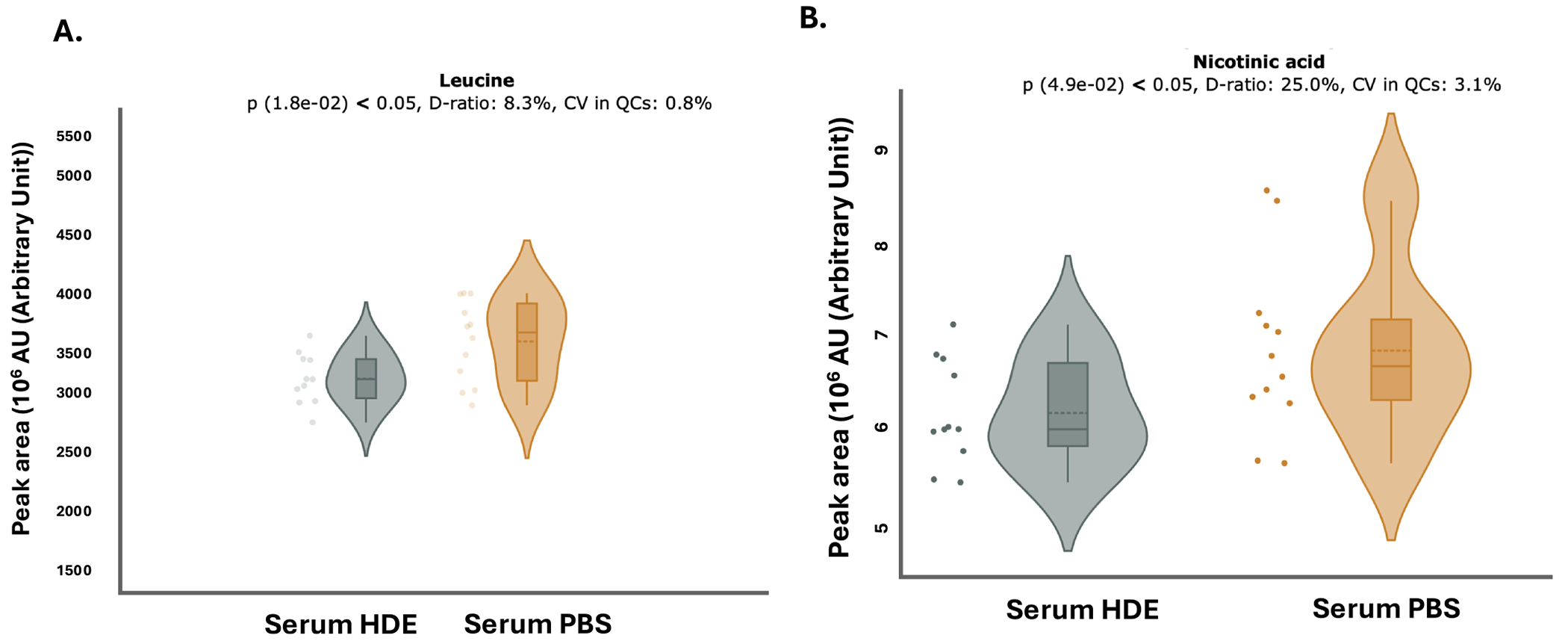
HDE exposure alters micronutrients and amino acids involved in energy metabolism. Metabolomic analysis found HDE-exposed mice have decreased leucine (Figure 9A; *p* = 0.018, *n* = 11) and nicotinic acid (Figure 9B; *p* = 0.049, *n* = 11) compared to saline controls.

**FIGURE 10 |:**
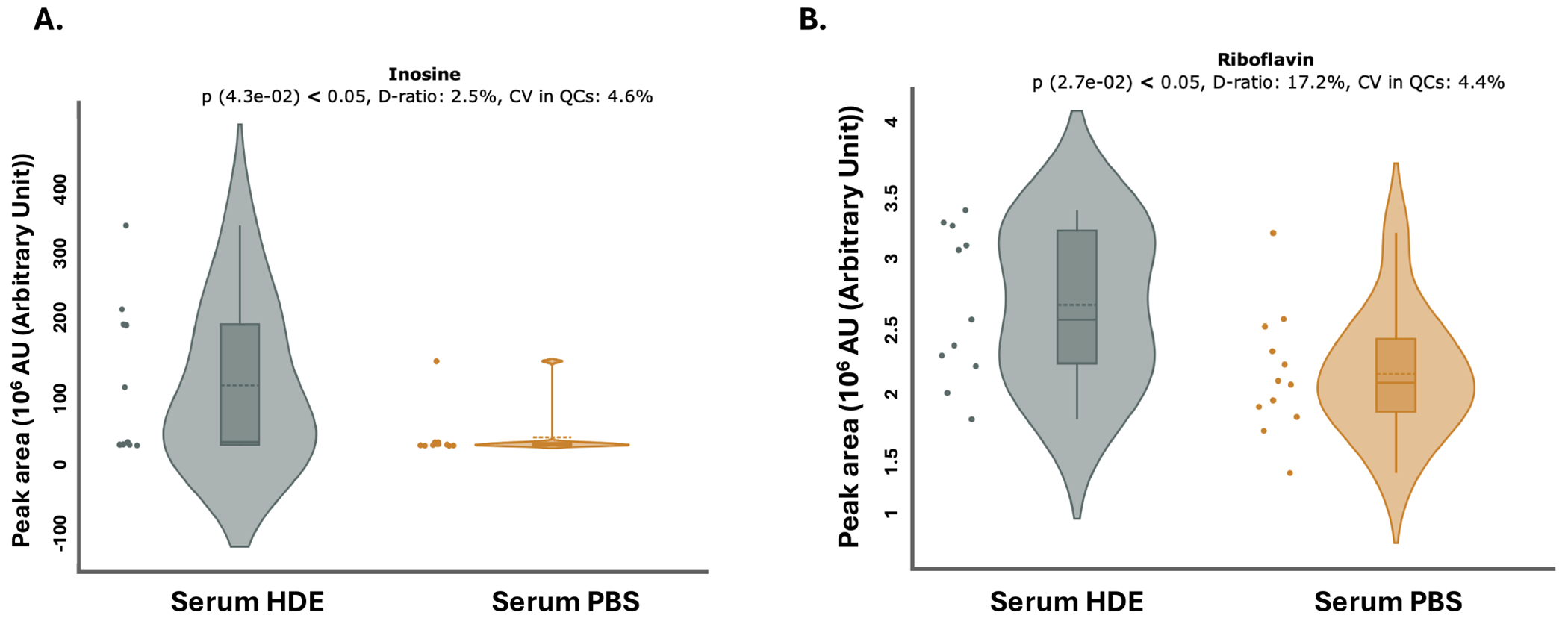
HDE exposure increases the abundance of essential micronutrients and amino acids. Metabolomics analysis found HDE-exposed mice have a significant increase in inosine (Figure 10A; *p* = 0.043, *n* = 11) and in riboflavin (Figure 10B: *p* = 0.027, *n* = 11).

Given the observed fluctuations in *A. muciniphila* and the alterations detected in metagenomic profiles, examining shifts in the metabolite landscape is a critical step to determine how alterations in gut microbial communities translate to functional metabolic changes. Serum metabolomic analyses revealed that HDE exposure reduced the expression of key metabolites involved in energy metabolism, leucine (Figure 9A, *p* = 0.018, *n* = 11) and nicotinic acid (Figure 9B, *p* = 0.049, *n* = 11). Additionally, HDE increased the expression of the essential amino acid, inosine (Figure 10A, *p* = 0.043, *n* = 11), and riboflavin (Vitamin B2) (Figure 10B, *p* = 0.0270, *n* = 11).

## 4 | Discussion

This study explores the effects of acute intranasal exposure to aqueous agricultural dust on developing gut dysbiosis. We hypothesize that such exposure would exacerbate intestinal permeability and gut barrier function. We found that repeated exposure to HDE did not alter the relative abundance of gut microbial communities, indicating a potential resilience of gut microbiota to acute agricultural pollutant exposure. This finding contrasts with existing literature suggesting that environmental pollutants, whether inhaled or intragastrically administered, generally disrupt microbial balance (Singh et al. 2022). This may imply a unique adaptive capacity of the gut microbiota in response to HDE exposure and indicate selective effects of different environmental pollutants on gut microbe communities.

We also observed an increase in alpha diversity within HDE-exposed mice, as measured by Pielou’s evenness indices and Faith’s PD. The increase in evenness may reflect a transient response to HDE exposure indicating microbial species are present in roughly equal abundance. Additionally, an unchanged beta diversity suggest that community composition remains stable despite exposure to HDE. These results imply that HDE exposure does not dramatically shift microbial communities but may influence microbial function and host interactions.

Our study found that HDE exposure caused subtle shifts in the gut barrier protective species *A. muciniphila*. In our previous study (Crawford et al. 2024), HDE-treated mice showed elevated LPS serum levels indicative of systemic endotoxemia. Increased serum LPS has profound inflammatory effects on intestinal barrier function via alterations in microbial communities and epithelial cell function (Li et al. 2023). The localized reduction in mRNA expression of *A. muciniphila* in the proximal colon of HDE-exposed mice correlates with our observations of increased intestinal permeability (Crawford et al. 2024). The lack of *A. muciniphila* can increase permeability through several mechanisms, including altered mucus and short-chain fatty acid (SCFA) production (e.g., propionate and acetate) and epithelial cell proliferation (Desai et al. 2016). Moreover, diminished levels of *A. muciniphila* have been associated with various metabolic disorders (Zhou et al. 2021). In our study, reduced *A. muciniphila* may imply the potential for gut barrier dysfunction and the subsequent development of systemic inflammation following acute HDE exposure. Metagenomic analysis further supported these findings, revealing reduced levels of several other beneficial taxa, including certain *Lachnospiraceae* species. The observed changes in microbial composition may contribute to the reduced levels of key metabolites and amino acids detected in serum samples.

### 4.1 | Altered Metabolite Profile

Certain metabolites serve as intermediates for SCFA production and the regulation of lung and gut homeostasis (Fusco et al. 2023; Shastak and Pelletier 2023). In the lungs, riboflavin and nicotinic acid are integral to cellular metabolism and other processes that are vital for cellular functions in the respiratory epithelium and immune cells (Li, Chen, et al. 2024; Shastak and Pelletier 2023). Altering vitamin production modifies cellular repair mechanisms and antioxidant defenses which increases susceptibility to oxidative stress and tissue damage (Li, Ren, et al. 2024; Shastak and Pelletier 2023). Inosine modulates immune responses and has been shown to exert protective effects against pulmonary inflammation (Kim et al. 2022). Its increase in HDE-exposed mice might, therefore, be an anti-inflammatory and protective response in the lungs (Liaudet et al. 2001; Mao et al. 2022).

Additionally, leucine is critical for protein synthesis and regulating immune functions via the mTOR signaling pathway (Yang et al. 2023). Its loss can disrupt the synthesis of structural proteins, weaken the pulmonary epithelium and immune responses (Das et al. 2014). Moreover, these specific changes in metabolite levels can impact lung homeostasis, making the lungs more vulnerable to inflammatory diseases and reducing their capacity to respond effectively to environmental irritants.

Concerning the gut, the altered levels of *A. muciniphila* in mice exposed to HDE may coincide with observed alterations in gut-derived metabolites: riboflavin, nicotinic acid, inosine, and leucine. *A. muciniphila* is primarily known for its role in degrading mucin to maintain intestinal barrier integrity (Lukovac et al. 2014). Its reduced levels in HDE-exposed mice may impact the production of SCFAs, which other microbes utilize for metabolism and immune function (Rodrigues et al. 2022). Additionally, reduced *A. muciniphila* may have contributed to the observed increase in gut permeability and the subsequent translocation of LPS into systemic circulation of HDE-exposed mice (Crawford et al. 2024). This can potentially exacerbate inflammatory responses within the gut and other metabolically active organs. Such dysregulation could contribute to lung inflammation, highlighting a critical interaction between gut microbial dynamics and lung health (Enaud et al. 2020). In our study, the associated decrease in metabolites like leucine and nicotinic acid, both essential for cellular energy metabolism, may further impair tight junction regulation and barrier integrity via reactive oxygen species pathways (Song et al. 2020; Lin et al. 2022). In addition to the decrease in *A. muciniphila*, other beneficial microbes essential to metabolite production, such as *Clostridium sp. ASF356, Lachnospiraceae* bacterium, and *Anaerotruncus* species were reduced in HDE fecal samples. Although the role(s) of *Anaerotruncus* species in the gut has not been thoroughly investigated, studies have found that these species are associated with nucleoside metabolism and amino acid fermentation (Raimondi et al. 2021). Therefore, reduced *Anaerotruncus* abundance may impact the fermentation of dietary fibers and proteins important for epithelial barrier function (Fu et al. 2023). *Clostridium* species are important for branched-chain amino acid (BCAA) metabolism (Ren et al. 2024). These species can convert leucine into SCFAs like isobutyrate and isovalerate, which are important for water and electrolyte balance in the colon (Mager et al. 2020). Moreover, *Lachnospiraceae* members have been shown to participate in BCAA metabolism, thereby influencing energy homeostasis (Li, Chen, et al. 2024). Both *Clostridium* and *Lachnospiraceae* are known to participate in nicotinic acid (niacin) metabolism, and a reduction in these bacteria could decrease niacin availability and promote intestinal inflammation and barrier dysfunction (Karunaratne et al. 2020), which aligns with the inflammatory effects of HDE exposure.

In our study, inosine and riboflavin increased following HDE exposure. Inosine regulates intestinal barrier function by reducing inflammation and acting on the peroxisome proliferator-activated receptor (PPAR)-y signaling pathway, prompting goblet cell and mucus production (Tang et al. 2024). Studies have also indicated riboflavin as an important regulator of tight junction expression in the intestine and a mediator of intestinal inflammation, possibly due to its antioxidant functions (Zhang et al. 2024; Xu et al. 2022). *Lachnospiraceae* bacteria and *Clostridium* species are involved in the breakdown and/or production of inosine via bacterial nucleosidases (purine metabolism) and riboflavin biosynthesis and uptake (LeBlanc et al. 2013; Magnúsdóttir et al. 2015). Although these bacteria decreased, an increase in metabolites may demonstrate a protective response against HDE exposure. Additionally, the increased riboflavin found in HDE-treated mice may mitigate intestinal epithelial cell permeability by influencing the transcription of tight junction genes (Chen et al. 2015). Moreover, riboflavin deficiency can impact the production of other metabolites by interrupting riboflavin kinase activity, which phosphorylates riboflavin, a precursor to flavin mononucleotide (FMN) and flavin adenine dinucleotide (FAD) production (Chengalroyen et al. 2024; Moreno et al. 2022). Therefore, increased riboflavin in HDE mice may also represent a compensatory effect to prevent the loss of other metabolites. This possible interplay between microbial alterations, metabolite dysregulation, and immune response underscores the complex mechanisms through which environmental factors like HDE may influence both gut and lung health, potentially driving inflammatory processes.

Significant reductions in *Lachnospiraceae* bacterium A2 and M18 were observed in the gut microbiota of mice exposed to HDE. Members of the *Lachnospiraceae* family are known to play pivotal roles in the fermentation of dietary fibers, producing SCFA and other metabolites critical for intestinal homeostasis and metabolic balance. More specifically, *Lachnospiraceae* are involved in the metabolic processes that contribute to the biosynthesis of B vitamins and amino acids, essential components for cellular energy metabolism and protein synthesis (Vacca et al. 2020).

The reduction in these specific *Lachnospiraceae* strains may disrupt metabolic pathways, decreasing the production of these critical metabolites and exacerbating the physiological responses observed in HDE-exposed mice. This association highlights the link between dietary fiber fermentation by the gut microbiota and metabolite production, suggesting that HDE can alter microbial communities and their metabolic functions. The gut microbiomes’ ability to modulate systemic immunity and inflammation through metabolic intermediates highlights the potential of microbial and metabolic alterations to impact gut and lung function under inflammatory conditions like those induced by HDE exposure.

## 5 | Conclusion

Pollutants generated from agricultural productions increase the risk of gut dysbiosis, especially among farmers employed in AFO (Shang et al. 2022). The findings from our intranasal exposure model identified novel effects of an agricultural dust extract on both gut bacterial population dynamics and metabolic capabilities that provide clues to the pathophysiological impact of dust exposure. The stability of the gut microbiome, subtle reductions in beneficial microbes, and alterations in metabolic profiles highlight the complexity of host–microbe interactions in the context of environmental exposures. Continued investigation of the long-term consequences of such exposures and the potential for therapeutic interventions targeting the gut microbiota is imperative to mitigating the health effects of agricultural pollutants.

### 5.1 | Study Limitations

The study focused on a relatively short exposure period (3 weeks). However, a longer duration may reveal different effects on microbial communities and systemic health. Additionally, a single time point analysis of the microbiome (at Day 15) may have excluded dynamic changes in the microbiota that occur over time. Moreover, the serum metabolomics findings (systemic) do not represent the potential localized gut changes (fecal metabolomics). Finally, while microbial composition was analyzed, functional implications (e.g., metabolic activity and immune response) of the observed changes were not directly assessed.

## Data Availability

The data that support the findings of this study are available from the corresponding author upon reasonable request.
